# Editorial: Innovative approaches to neuralgia: mechanisms and treatment development

**DOI:** 10.3389/fneur.2026.1832495

**Published:** 2026-04-09

**Authors:** Wangjun Qin, Peng Mao, Yanting Wang, Guijun Lu, Junxu Li

**Affiliations:** 1 Department of Pharmacy, China-Japan Friendship Hospital, Beijing, China; 2 Department of Pain Management, China-Japan Friendship Hospital, Beijing, China; 3 Department of Pharmacy, National Cancer Center/National Clinical Research Center for Cancer/Cancer Hospital, Chinese Academy of Medical Sciences and Peking Union Medical College, Beijing, China; 4 Palliative Care Department & Pain Department, Beijing Tsinghua Changgung Hospital, Beijing, China; 5 Department of Pharmacology and Toxicology, University at Buffalo, Buffalo, NY, United States

**Keywords:** mechanisms, neuralgia, neuropathic pain, post-herpetic neuralgia, therapeutic strategies

Neuralgia represents a debilitating class of neuropathic pain disorders characterized by paroxysmal pain along nerve pathways, with a disproportionate impact on older populations and a heavy burden on individual quality of life and healthcare systems worldwide (1, 2). Despite its high clinical prevalence, the pathophysiological mechanisms underlying most neuralgia subtypes remain incompletely elucidated, and clinical management is often limited by suboptimal therapeutic efficacy, poor treatment specificity, and the lack of targeted interventions (3). This Research Topic, *Innovative approaches to neuralgia: mechanisms and treatment development*, aims to address these critical gaps by presenting cutting-edge research into the molecular, cellular, and clinical mechanisms of neuralgia, alongside novel diagnostic, prognostic, and therapeutic strategies across multiple neuralgia subtypes, including post-herpetic neuralgia (PHN), trigeminal neuralgia (TN), diabetic peripheral neuropathic pain, and other refractory neuropathic pain conditions. The collection of studies within this Topic spans systematic reviews, original research, study protocols, and bibliometric analyses, collectively advancing our understanding of neuralgia and providing actionable clinical and translational insights for its management.

A core focus of this Research Topic is the advancement of predictive models for neuralgia, a critical step toward early intervention and risk stratification. A systematic review and meta-analysis evaluating machine learning for PHN prediction demonstrated excellent diagnostic performance of these models (sensitivity: 0.81, specificity: 0.84, AUC: 0.90), while identifying dataset source, model selection, and predictor choice as key sources of heterogeneity (Lin et al.). This work highlights the potential of artificial intelligence in PHN prognostication, while also emphasizing the need for strict external validation and standardized reporting to address overfitting and limited generalizability—key limitations of current predictive models. Complementary original research identified age, pain severity at onset, C-reactive protein (CRP), and homocysteine (HCY) as independent risk factors for PHN in immunocompetent herpes zoster patients, with a combined predictive model achieving 68.4% sensitivity and 74.1% specificity (Liu et al.). These findings establish serum CRP and HCY as promising non-invasive biomarkers for PHN, filling a critical gap in prognostic tools for immunocompetent individuals, a population historically understudied in PHN research.

For TN, a leading and highly disabling neuralgia subtype (4), this Research Topic presents multiple studies advancing both mechanistic understanding and therapeutic optimization. Histopathological research revealed significant arachnoid fibrosis and thickening in the cerebellopontine angle of TN patients, driven by increased collagen fiber deposition, providing a novel pathological mechanism underlying TN pathogenesis and a potential new target for therapeutic intervention (Tang et al.). Clinical studies on TN treatment further refined existing approaches. A long-term follow-up study of Gamma Knife radiosurgery (GKS) demonstrated that biologically effective dose (BED) modulates treatment efficacy and safety in a target location-dependent manner, with BED adjustment for distal targets optimizing clinical outcomes (Deng et al.). Comparative research on microvascular decompression (MVD) for TN found endoscopic and microscopic approaches to yield comparable clinical efficacy, while endoscopy offered superior minimally invasiveness, more accurate identification of offending vessels, and higher rates of full trigeminal nerve exposure, which provides insights that inform personalized surgical planning for TN patients (Li L. et al.). Additionally, pilot research on sphenopalatine ganglion (SPG) stimulation showed promising long-term pain relief for refractory trigeminal neuropathic pain, offering a novel neuromodulatory alternative for patients unresponsive to conventional neurosurgical treatments (Sokal et al.).

Refractory neuralgia, particularly PHN, remains a major clinical challenge (5), and this Research Topic features several innovative interventional and pharmacological treatment strategies with robust clinical evidence. A 1-year follow-up study confirmed CT-guided intervertebral foramen ozone injection (IVFO) as a safe and effective approach for chronic, drug-resistant thoracic and lumbar PHN, with significant and sustained pain relief, reduced mechanical hyperalgesia, and nearly 50% of patients discontinuing gabapentin within 3 months (Wang J.-L. et al.). Retrospective research on continuous epidural anesthetic and steroid infusion for acute herpes zoster demonstrated superior efficacy in accelerating skin healing, relieving acute pain, and reducing PHN incidence (11.2% vs. 23.8%) compared to conventional oral therapy, with comparable safety and higher patient satisfaction (Dou et al.). For perineal herpes zoster pain—a rare and difficult-to-treat subtype—ultrasound combined with DSA-guided pulsed radiofrequency (PRF) was shown to shorten puncture time, reduce hematoma risk, and improve procedural satisfaction, while PRF itself effectively alleviated pain and central sensitization (Dong et al.). Furthermore, a randomized, double-blind, placebo-controlled multicenter study protocol for interferon-α1b intervertebral foramen injection in PHN provides a roadmap for evaluating a novel targeted biological therapy, addressing the unmet need for evidence-based biological interventions in refractory PHN (Hu et al.).

Beyond clinical translation, this Research Topic also advances mechanistic insights into neuralgia pathogenesis at the molecular and cellular levels. Original research identified six m1A/m6A/m5C/m7G RNA methylation-related genes (Fto, Mettl3, Nsun2, Ythdf3, Wdr4, Eif4e) as differentially expressed in neuropathic pain, with two distinct RNA methylation clusters linked to MAPK and FoxO signaling pathway activation, which establishes RNA epigenetic modification as a novel regulatory mechanism in neuralgia and a potential new source of therapeutic targets (Tan et al.). Bibliometric analyses further contextualize the global research landscape. One study mapped the role of glial cells in neuralgia, identifying glial cell activation, neuroinflammation, and oxidative stress as core research hotspots (He et al.), while another analyzed spinal cord injury-related neuropathic pain research, highlighting signaling pathways, spinal cord stimulation, and comorbid depression as emerging focus areas (Huiqing et al.). These bibliometric studies not only summarize the current state of neuralgia mechanistic research but also guide future investigative directions for the field.

The Research Topic also addresses critical comorbidities and risk factors that modulate neuralgia presentation and outcomes, as well as understudied pain subtypes. A meta-analysis of painful diabetic peripheral neuropathy (PDPN) established a 33.9% global prevalence and identified female gender, glycemic control, microvascular complications, and lifestyle factors (smoking, obesity) as key risk factors, providing a basis for early risk stratification and preventive intervention in diabetic patients (Zhou et al.). A cross-sectional study on migraine, which often comorbid with neuralgia, found reduced self-esteem and increased anxiety/depression in migraine patients, with self-compassion declining with higher attack frequency, emphasizing the need for integrated psychological support in pain management (Karakurum-Goksel et al.). For other refractory pain conditions, research on radiofrequency ablation of the sinuvertebral nerve demonstrated excellent short-term efficacy for discogenic low back pain after lumbar interbody fusion (Wang L. et al.), while a case report of multi-regional sequential pain syndrome (MRPS) identified cryptogenic vertebral artery dissection as a key cause, expanding the clinical spectrum of pain disorders linked to vascular pathology (Yang X. et al.). Additionally, a systematic review evaluated the effectiveness of dry needling for plantar fasciitis, adding to the evidence base for non-surgical pain interventions (Yang A. et al.), and a meta-analysis of refractory bone metastasis pain identified multiple bone metastases and lytic lesions as key risk factors, addressing pain management in oncological populations (Li Q. et al.). A cross-sectional study also uncovered an S-shaped association between dietary caffeine intake and severe headache or migraine, providing dietary insights for pain prevention (Liao et al.).

Collectively, the studies in this Research Topic advance neuralgia research across mechanistic, prognostic, and therapeutic domains, filling critical gaps in our understanding of understudied populations (e.g., immunocompetent PHN patients), refining existing clinical interventions, and presenting novel targeted strategies for refractory disease. Key takeaways include the potential of machine learning and serum biomarkers for early neuralgia prediction, the importance of personalized therapeutic approaches (e.g., location-dependent GKS dosing for TN, minimally invasive endoscopic MVD), and the promise of interventional and biological therapies for drug-resistant PHN. Additionally, these studies highlight the need for standardized reporting in predictive model development, large-scale prospective trials for novel interventions, and integrated management of neuralgia and its psychological comorbidities. As neuralgia research continues to evolve, the insights from this Research Topic provide a foundation for translational progress, with the ultimate goal of developing more targeted, effective, and safe treatments for patients with this debilitating disorder (6). Moving forward, interdisciplinary research integrating molecular biology, artificial intelligence, neurosurgery, and pain medicine will be critical to further unraveling neuralgia mechanisms and translating these findings into improved clinical outcomes.
